# Whole genome expression profiling of blood cells in ovarian cancer patients -Prognostic impact of the *CYP1B1*, *MTSS1*, *NCALD*, and *NOP14* genes

**DOI:** 10.18632/oncotarget.1938

**Published:** 2014-05-01

**Authors:** Helena S. Isaksson, Bengt Sorbe, Torbjörn K. Nilsson

**Affiliations:** ^1^ School of Health and Medical Sciences, Örebro University, Sweden; ^2^ Department of Oncology, Örebro University Hospital, Sweden; ^3^ Department of Medical Biosciences/Clinical Chemistry, Umeå University,Umeå, Sweden

**Keywords:** ovarian cancer, whole genome profiling, prognosis, mRNA, NCALD, MTSS1, PDA3, CYP1B1, NOP14, LYAR

## Abstract

Ovarian cancer patients with different tumor stages and cell differentiation might be distinguished from each other by gene expression profiles in whole blood cell mRNA by the Affymetrix Human Gene 1.0 ST Array. We also examined if there is any association with other clinical variables, response to therapy, and residual tumor burden after surgery. Patients were divided into two groups, one with poor prognosis, advanced stage and poorly differentiated tumors (n = 22), and one group with good prognosis, early stage and well- to medium differentiated tumors (n = 11). Six genes were found to be differentially expressed: the *PDIA3, LYAR, NOP14, NCALD* and *MTSS1* genes were down-regulated and the *CYP1B1* gene expression was up-regulated in the poor prognosis group, all with p value <0.05, adjusted for mass comparison. In survival analyses, *CYP1B1, MTSS1, NCALD* and *NOP14* remained significantly different (p<0.05). Patient groups did not differ in any transcript related to acute phase or immune responses. This minimal gene expression signature of prognostic ovarian cancer-related genes opens up an avenue for more practicable monitoring of ovarian cancer patients by simple peripheral blood tests, which may evolve into a tool to guide selection of curative and postoperative supportive therapies.

## INTRODUCTION

Ovarian cancer is an important disease among the gynecological malignancies. Despite a slowly decreasing incidence in many Western countries the prognosis is still unfavorable [1], and the overall 5-year survival rate is approximately 50% at the best centers after primary cytoreductive surgery and combination chemotherapy with paclitaxel and carboplatin [2]. Significant improvements in treatment results have been achieved during the last decades and further improvements can be expected in the future for this disease. Many clinical trials are ongoing to improve chemotherapy, but also to incorporate target therapy agents [2,3].

Predictive and prognostic factors are important in guidance of expected response and survival and for the choice of optimal primary therapy [4,5]. A number of prognostic factors identified so far are mainly clinical, e.g. stage, type of histology, FIGO grade, and residual tumor after primary surgery [6,7]. The amount of residual tumor is in fact among the strongest prognostic factors for survival [6,7]. The goal of the primary cytoreductive surgery is to reduce the tumor volume as much as possible to no residual tumor macroscopically or at least to less than 1 cm tumor diameter of the remaining nodules. Centralized surgery and experienced tumor surgeons are important to achieve this goal, but the biology of the individual tumor is also thought to be of importance for the outcome of the surgery and prognosis [6,8]. It should therefore be possible to identify biomarkers in a blood sample that adds prognostic value, and an alternative to performing a biopsy of tumor tissue.

The biology of individual ovarian tumors can be characterized by their genetic profiles with up- or down-regulation of important oncogenes and tumor suppressor genes. DNA changes and expression of RNA can be studied with microarray techniques on tissue samples from the tumor. Fresh or fresh-frozen tissue is generally needed for these analyses, but often these types of specimens are not available in the routine clinical work, especially during postoperative follow-up. A more practicable way would be to analyze blood cell samples from the individual patient, both blood leukocytes and circulating tumor cells may be the sources of mRNA in these analyses [9,10], but on a molar basis the leukocytes can be expected to be the dominating source of mRNA. The mRNA species from leukocytes is thought to reflect more general and systemic reactions and tumor cell mRNA species would reflect specific tumor characteristics. In our pilot study, we corroborated that two groups of ovarian cancer patients with or without residual tumor mass after primary surgery showed differences in gene expression profiles in blood cells which seemed to agree with such a contention since most of the genes that differed belonged to rather cancer-specific pathways [11]. In the present study, we therefore tested the hypothesis that patients with different tumor stage and cell differentiation can be distinguished from each other by performing a whole transcriptome profile in whole blood cell mRNA of ovarian cancer patients. We also wished to examine if these profiles were associated with other clinical variables, such as therapy response, survival and residual tumor burden after surgery.

## RESULTS

### Clinical characteristics

The characteristics of the patients and tumors are presented in Table 1. The complete series analyzed encompassed 33 patients with ovarian carcinomas (FIGO stages I-IV), pre-selected to represent a high-risk group (Group A, n=22) and a low-risk group (Group B, n=11). FIGO stage (stage III-IV vs. I-II) and tumor grade (grade 3 vs. grade 1-2) were used to define the two groups. The mean age of the patients in the two risk groups (63.6 and 60.3 years) was not significantly different. All tumors included were adenocarcinomas. In the high-risk group 21 of 22 cases (95.5%) were seropapillary adenocarcinomas, and in the low-risk group seven of 11 cases (63.6%). In the latter group two tumors were of the endometrioid type and two cases were clear cell carcinomas. This difference was statistically significant (p = 0.016). Residual carcinoma after the primary surgery was more frequent in the high-risk group (68.2%) than in the low-risk group (18.2%), p = 0.007. The mean follow-up period for patients alive was 42.1 months (range 14-86 months). The 5-year overall survival rate of the complete series was 48.8% (95% CI 28.4-69.2%) and differed between the groups; in the high-risk group 28.8% and in the low-risk group 100% (log-rank test; p = 0.0004).

**Table 1:** Patient, tumor and surgery characteristics of the two subgroups

	Group A	Group B	p value
Patients (n)	22	11	
Age (mean, SD)	63.6 (11.9)	60.3 (10.5)	0.440
			
FIGO stage (n)			
IA	0	6	
IB	0	1	
IC	0	1	
IIC	0	2	
IIIB	1	0	
IIIC	21	0	
IV	0	1	
Histology (n)			
Seropapillary (1c)	21	7	
Endometrioid type (3c)	0	2	
Clear cell carcinoma (4c)	0	1	
Mixture of 1c and 4c	1	1	
Tumor grade (n)			
Grade 1	0	4	
Grade 2	0	7	
Grade 3	22	0	
Residiual tumor (n)			
No residual carcinoma (0 cm)	7 (31.8%)	9 (81.8%)	
Residual carcinoma (> 0 cm)	15 (68.2%)	2 (18.2%)	
			
Surgery (n)			
TAHBSO	16	9	
TAHUSO	0	1	
BSO	2	1	
USO	2	0	
Laporatomy and biopsy	2	0	

TAHBSO: Total abdominal hysterectomy and bilateral salpingo-oophorectomy; TAHUSO: Total abdominal hysterectomy and unilateral salpingo-oophorectomy; BSO: Bilateral salpingo-oophorectomy; USO: Unilateral salpingo-oophorectomy.

P value calculated by t-test.

### Gene expression data as predictors of outcome

**Figure 1:**
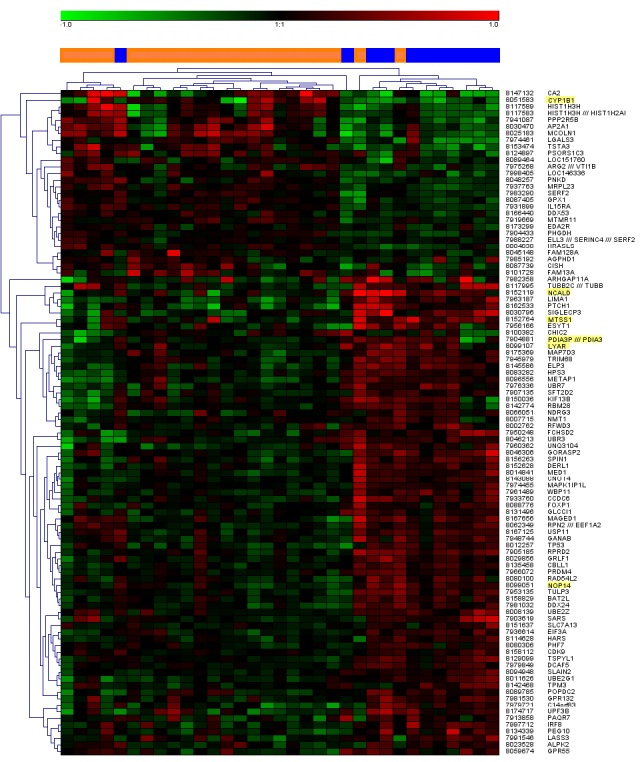
Unsupervised cluster analysis of the top 100 most differentially regulated genes In the center bar, orange color denotes patient samples from group A and blue color denotes samples from group B. Gene expressions with statistically significant corrected p values are highlighted in the gene name list to the right.

An unsupervised cluster analysis was made from the gene expression array for the 100 genes with lowest unadjusted p values including all patients from groups A and B, whereby only three patients were misclassified (see heat map in Figure 1). Six genes; *PDIA3, CYP1B1, LYAR, NOP14, NCALD* and *MTSS1* were found to be expressed significantly different between the two groups when adjusted for multiple testing (Table 2).

**Table 2:** Blood leukocyte gene expression profiles of ovarian cancer patients, unguided analysis Comparison of Group B vs. Group A, a negativ fold change indicates a down-regulation of gene expression. The moderated t-statistics generated the p value in the same manner as an ordinary t-test. Adjusted p value is also known as q-value or FDR. This is a Benjamini and Hochberg's method to control false positives

Probe Set ID	Gene Title	Gene Symbol	Log2 Fold Change	p value	Adj. p value
7904881*	protein disulfide isomerase family A, member 3	PDIA3P / PDIA3	0.271	1.31E-06	0.027
8051583	cytochrome P450, family 1, subfamily B, polypeptide 1	CYP1B1	- 0.507	1.9E-06	0.027
8099107	Ly1 antibody reactive homolog (mouse)	LYAR	0.313	3.09E-06	0.029
8099051	NOP14 nucleolar protein homolog (yeast)	NOP14	0.273	1.06E-05	0.050
8152119	neurocalcin delta	NCALD	0.400	9.68E-06	0.050
8152764	metastasis suppressor 1	MTSS1	0.369	1.05E-05	0.050

* This probe set contains probes for both the pseudogene and for the PDIA3 mRNA.

**Figure 2:**
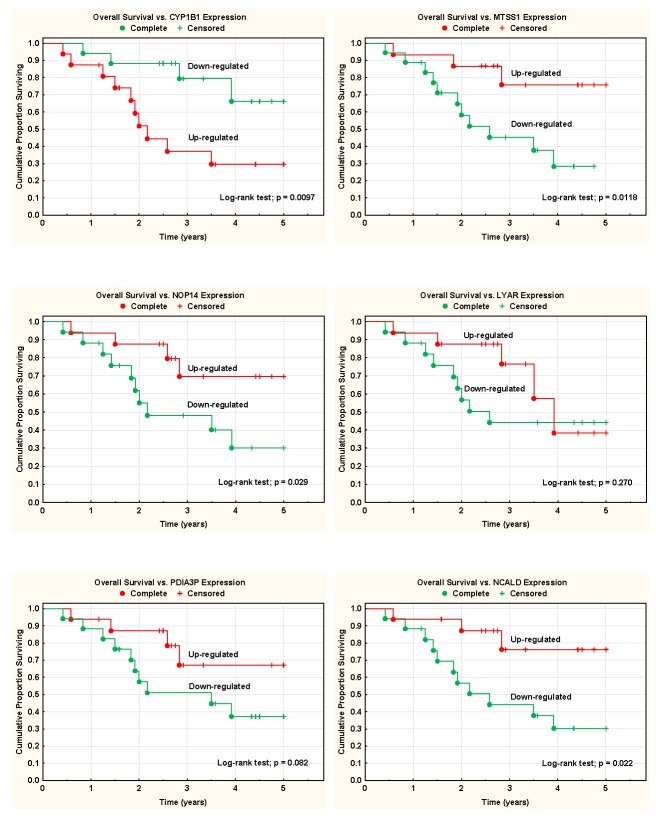
Survival analyses and blood mRNA expression data Overall survival rate was calculated for patients with leukocyte mRNA up-regulated (level above the median value of all patients) or down-regulated (level below the median) of the six genes analyzed.

At the time of analysis 15 patients (all in the high-risk group) were dead of disease. No cases of intercurrent death were recorded. Overall survival rate was calculated for patients with leukocyte mRNA up-regulated (level above the median value of all patients) or down-regulated (level below the median) of the six genes analyzed. Up-regulation of the *CYP1B1*-gene, and down-regulation of *MTSS1, NCALD*, and *NOP14* genes, was associated with a significantly inferior survival rate (Figure 2). Expression of PDIA3P and LYAR showed the same pattern, but the differences were non-significant.

The *CYP1B1*-gene was up-regulated (> median value) in 68% of high-risk tumors, but in only 9% in low-risk tumors (p = 0.0014). The *MTSS1* gene was down-regulated (< median value) in 77% of high-risk tumors and in 9% in low-risk tumors (p = 0.0002). The *NOP14, NCALD, PDIA3P* and *LYAR* were also all significantly down-regulated (68-73%) in the high-risk group (Table 3).

**Table 3:** Gene expression in the two predefined risk groups

Gene expression	High-risk group (A)	Low-risk group (B)	Χ2
CYP1B1 (> median)	68.2%	9.1%	0.0014
MTSS1 (< median)	77.3%	9.1%	0.0002
NOP14 (< median)	72.7%	9.1%	0.0006
NCALD (< median)	72.7%	9.1%	0.0006
PDIA3P (< median)	68.2%	18.2%	0.0067
LYAR (< median)	68.2%	18.2%	0.0067

There was a highly statistically significant association between tumor stages (stage I-II vs. III-IV) and expression of all six genes studied. Down-regulation of *MTSS1* was noted in 74% of advanced stage tumors, but only in 10% in early stages (p = 0.0007). *CYP1B1* was overexpressed in 65% of advanced stage tumors and in 10% in early stages (p = 0.0035). The other four genes were all significantly down-regulated in advanced stages (Table 4).

**Table 4:** Gene expression and tumor characteristics

Gene expression	Stage III-IV	Stage I-II	Χ2	Grade 3	Grade 1-2	Χ2	Serous type	Non-serous type	Χ2
CYP1B1 (> median)	65.2%	10.0%	0.0035	68.2%	9.1%	0.0043	53.6%	20.0%	0.1665
MTSS1 (< median)	73.9%	10.0%	0.0007	77.3%	9.1%	0.0009	60.7%	20.0%	0.0922
NOP14 (< median)	69.6%	10.0%	0.0017	72.7%	9.1%	0.0024	53.6%	40.0%	0.5759
NCALD (< median)	69.6%	10.0%	0.0017	72.7%	9.1%	0.0024	57.1%	20.0%	0.1258
PDIA3P (< median)	69.6%	10.0%	0.0017	68.2%	18.2%	0.0240	57.1%	20.0%	0.1258
LYAR (< median)	69.6%	10.0%	0.0017	68.2%	18.2%	0.0170	57.1%	20.0%	0.1258

Serous papillary carcinomas were most frequent in this series and this type of histology showed borderline association with expression of *MTSS1* (p = 0.092). For the other five gene types this association was not statistically significant.

On the other hand, FIGO-grade of the tumor was highly statistically associated with expression of all six genes. FIGO-grade 3 was compared with FIGO-grade 1-2 in the analyses. *MTSS1* showed the strongest association with poorly differentiated tumors, and 77% of these tumors showed down-regulation of this gene (Table 4).

A statistical model using Cox proportional regression analysis and the best subset technique showed that a combination of the up-regulated *CYP1B1* and the down-regulated *MTSS1* gene expressions predicted overall survival rate most efficiently. A three-gene model also included *NOP14*. Addition of information from the other genes only marginally improved the model.

## DISCUSSION

In this whole genome expression study on blood cell mRNA from ovarian cancer patients, only six genes, *PDIA3*, *CYP1B1*, *LYAR*, *NOP14*, *NCALD,* and *MTSS1* showed a statistically significant difference in expression between subjects with tumors that were poorly differentiated vs. those who had moderately to well differentiated tumors. Four of these, *CYP1B1*, *NCALD, NOP14,* and *MTSS1* C were significantly associated with prognosis in survival analyses (Figure. 2). Since tumor differentiation is a major prognostic factor, it makes sense that these genes account partly for this difference in prognosis. This is further supported by the known functions of the six genes, which all appeared to be of relevance for tumor biology in general, and in particular for a partly estrogen-linked tumor such as ovarian cancer, as outlined below. In a cluster analysis based on the gene expression data, only three of the 33 included patients were misclassified (Figure. 1).

The *CYP1B1* (Cytochrome P450, family 1, subfamily B, polypeptide 1) mRNA encodes a protein that catalyses reactions involved in drug metabolism and the synthesis of lipids, including cholesterol and steroids [12,13]. A search in the BioGPS database [14] confirmed gene expression in normal whole blood and in particular in CD14+ monocytes. The protein can be detected in several normal tissues as well as in tumor and metastasis tissues, levels tend to be elevated in tumor tissue compared to normal tissue [13]. Some studies reported it to be undetectable in normal tissue but detectable in tumor and metastasis tissue [12,15]. Importantly, *CYP1B1* can be found in tissues that are estrogen-stimulated, like the breast, ovary, and uterus [16]. In these tissues its main function is to catalyze the hydroxylation of estradiol to 4-hydroxyl estradiol (4-OHe2) [16]. Several studies have suggested that the *CYP1B1* gene may be a marker for ovarian cancer and a possible target for intervention [13,15,16]. Modugno et al argues that subgroups of ovarian cancer patients respond well to endocrine treatment and calls for biomarkers that can predict such patients [17]. Thus, it is remarkable and suggestive of some systemically active regulatory process that we could pick up a significant difference in mRNA levels of this particular gene between the two patient groups even in cells from peripheral blood.

The *MTSS1* (metastasis suppressor 1) gene, also known as Missing in Metastasis gene (*MIM*), encodes a protein that contains multiple functioning motifs, thought to act as an actin-binding scaffold protein. It has been implicated in carcinogenesis and metastasis; some researchers consider it to be a potential metastasis suppressor gene [18-20]. One study of colorectal cancer (CRC) found an increased *MTSS1* protein expression in CRC tissue compared to normal tissue and it was correlated to poor differentiation, tissue invasion, presence of lymph node metastases, high TNM stage: strong positive protein expression was associated with significantly shorter survival [19]. A loss of MTSS1 protein expression in gastric cancer has been associated with large tumor size, poor differentiation, deep invasion level, the presence of nodal metastasis, and poor outcome in patients who underwent gastrectomy [18]. The sparse clinical data is thus fairly contradictory. Animal and cell-line studies suggest that *MTSS1* is more resistant to cell-cell junction disassembly, and a loss of protein expression promotes epithelial-to-mesenchymal transition and metastasis [20,21]. Our results support the view that down-regulated blood cell *MTSS1* expression is a marker of worse prognosis in ovarian cancer.

The *NCALD* (neruocalcin delta) mRNA encodes a member of the neuronal calcium sensor (NCS) family of calcium-binding proteins. The protein is thought to be a regulator of G protein-coupled receptor signal transduction and several alternatively spliced variants of the gene exists, all encoding the same protein. *NCALD* gene expression can be found in several tissues [22], for example in many parts of the normal brain, natural killer cells, lymphoblasts, and trace amounts of *NCALD* gene expression can be found in healthy ovarian tissue [15]. So far very little is known about this gene in cancer. A study by Couvelard et al found *NCALD* gene expression to be one of many genes that can distinguish between metastatic and non-metastatic pancreatic endocrine tumor tissue [23]. However, another gene belonging to the same gene family, the neuronal Ca^2+^ sensor protein family (NCS), termed *VILIP1* [24], has been more extensively studied in cancer, and shown to act as a tumor suppressor gene by inhibiting cell proliferation, adhesion, and invasiveness [25,26]. The VILIP-1 protein and mRNA was down-regulated in a study on non-small cell lung carcinoma [25], and high gene expression was reported to be associated with a high rate of lymph node metastasis and poor prognosis in colorectal cancer patients [27].

PDIA3, the protein disulfide isomerase family A, member 3 gene, encodes a protein in the endoplasmatic reticulum that interacts with lectin chaperones calreticulin and calnexin to modulate the folding of glycoproteins that are newly synthesized [28,29]. The protein PDIA3 (also known as ERp57, GRP58, ERp60, and ERp61) has been found to be active in several other locations and reactions, for example interactions in the nucleus which involve DNA repair, DNA damage recognition, and apoptosis [28,29]. A study of a number of different ovarian cancer cell-lines reported *PDIA3* mRNA expression to be strongly elevated compared to human ovarian surface epithelial cells, and protein expression followed the same pattern [30]. Cicchillitti et al described that paclitaxel-resistant cells lack the normal interaction between b-actin and PDIA3 [29]. The BioGPS database [14] confirmed *PDIA3* gene expression in normal whole blood cells and most other tissues.

The Ly1 antibody reactive homolog (LYAR) was first described by Su et al as a cDNA encoding zinc finger protein isolated from mouse T-cell leukemia line, they also showed that cells with this protein had increased ability to form tumors in nu/nu mice and therefore called it a nucleolar oncoprotein in cell growth regulation [31]. The BioGPS database [14] showed that *LYAR* gene expression is found in many normal tissues and whole blood. Highest levels are reported in NK-cells, T-cells, lymphoblasts, CD34+ cells, and testis interstitial tissue.

Finally, we find it remarkable, and worth stressing, that no expression signature indicating unspecific disease activity in the immune system or general acute-phase inflammatory response mechanisms, such as that found in a recent study on prostate cancer [32], seemed to differentiate the poor and good prognosis groups. This raises our expectations that the novel prognostic signature described here is a real feature of the prognostic differences in tumor biology within the panorama of ovarian cancer.

In conclusion, we propose six genes that are promising candidates as a prognostic biomarker signature measured as mRNA in peripheral blood cells in ovarian cancer patients, *PDIA3*, *CYP1B1*, *LYAR*, *NOP14*, *NCALD,* and *MTSS1*. Monitoring of these in peripheral blood samples in future longitudinal multicenter follow-up studies, will be necessary for validation of the clinical utility of this proposed prognostic gene expression signature.

## MATERIAL AND METHODS

### Ethics statement

Investigation has been conducted in accordance with the ethical standards and according to the Declaration of Helsinki and according to national and international guidelines and has been approved by the authors' institutional review board, the Regional Board of Ethics, Uppsala, Sweden. Written informed consent was obtained from the patients.

### Subjects

Blood samples were consecutively collected from ninety-two women with ovarian cancer, FIGO (International Federation of Gynecology and Obstetrics) stage I-IV, admitted for treatment at the Department of Gynecological Oncology, University Hospital in Örebro, Sweden from October 2004 to December 2011. Enrollment took place 2-4 weeks after the primary cytoreductive surgery. Patients with a defined tumor stage and differentiation by a reference pathologist were considered for this project, and samples with RNA of satisfactory quality (see methods) were then analyzed. Thirty-three of the patients were included in this study. Patients were divided into two groups, A and B, one with a known poor prognosis; poorly differentiated tumors (n = 22), and one group with good prognosis; well- to medium well differentiated tumors (n = 11). See Table 1 for tumor characteristics.

### Blood collection and extraction

The blood was collected in PAXgene tubes and the total RNA was extracted with PAXgene Blood RNA Kit (QIAGEN Inc., Valencia, CA, USA) in compliance with the manufacturer's instructions. Total RNA concentration was measured with spectrophotometry on a ND-1000 instrument (NanoDrop Technologies, Wilmington, DE, USA) absorbance ratio (260/280 nm) between 1.9-2.2 accepted. RNA quality was evaluated on an Agilent 2100 Bioanalyzer (Agilent Technologies, Waldbronn, Germany), A RIN (RNA integrity number) over seven was considered as good quality.

### Gene expression analysis and statistical calculations

To generate biotinylated sense-strand cDNA, 250 ng of total RNA were used from each patient according to Ambion WT Expression Kit (P/N 4425209 Rev B 05/2009) and Affymetrix GeneChip® WT Terminal Labeling and Hybridization User Manual (P/N 702808 Rev. 1, Affymetrix Inc., Santa Clara, CA, USA). Samples were hybridized to a GeneChip® Human Gene 1.0 ST Array (Affymetrix Inc., Santa Clara, CA, USA) and scanned using the GeneChip®Scanner 3000 7G at the Uppsala Array Platform (Uppsala University, Sweden) according to the manufacturer's instructions. The raw data was normalized in the free software Expression Console provided by Affymetrix (http://www.affymetrix.com) using the robust multi-array average (RMA) method first suggested by Li and Wong in 2001 [33,34]. Subsequent analysis of the gene expression data was carried out in the freely available statistical computing language R (http://www.r-project.org) using packages available from the Bioconductor project (www.bioconductor.org). In order to search for the differentially expressed genes between the A and B groups an empirical Bayes moderated t-test was then applied [35], using the ‘limma’ package [36]. To address the problem with multiple testing, the p values were adjusted using the method of Benjamini and Hochberg [37]. SAS software packages were used for the statistical calculations.

Clinical characteristics were analyzed using Pearson's chi-square test, t-test, Kaplan-Meier survival analysis and log-rank test statistics. Cox proportional regression analysis and the best subset technique were used for prognostic modeling. A p value of 0.05 or less was regarded as statistically significant. Statistica software packages were used for the statistical calculations.
